# Contralaterally controlled neuromuscular electrical stimulation-induced changes in functional connectivity in patients with stroke assessed using functional near-infrared spectroscopy

**DOI:** 10.3389/fncir.2022.955728

**Published:** 2022-08-29

**Authors:** Chuan Guo, Youxin Sui, Sheng Xu, Ren Zhuang, Mingming Zhang, Shizhe Zhu, Jin Wang, Yushi Zhang, Chaojie Kan, Ye Shi, Tong Wang, Ying Shen

**Affiliations:** ^1^Department of Rehabilitation Medicine, The First Affiliated Hospital of Nanjing Medical University, Nanjing, China; ^2^department>School of Rehabilitation Medicine, Nanjing Medical University, Nanjing, China; ^3^Department of Rehabilitation Medicine, Changzhou Dean Hospital, Changzhou, China; ^4^Department of Psychology, Shanghai Normal University, Shanghai, China

**Keywords:** stroke, functional near-infrared spectroscopy, neuromuscular electrical stimulation, functional connectivity, upper extremity, wrist extension

## Abstract

Contralaterally controlled neuromuscular electrical stimulation (CCNMES) is an innovative therapy in stroke rehabilitation which has been verified in clinical studies. However, the underlying mechanism of CCNMES are yet to be comprehensively revealed. The main purpose of this study was to apply functional near-infrared spectroscopy (fNIRS) to compare CCNMES-related changes in functional connectivity (FC) within a cortical network after stroke with those induced by neuromuscular electrical stimulation (NMES) when performing wrist extension with hemiplegic upper extremity. Thirty-one stroke patients with right hemisphere lesion were randomly assigned to CCNMES (*n* = 16) or NMES (*n* = 15) groups. Patients in both groups received two tasks: 10-min rest and 10-min electrical stimulation task. In each task, the cerebral oxygenation signals in the prefrontal cortex (PFC), bilateral primary motor cortex (M1), and primary sensory cortex (S1) were measured by a 35-channel fNIRS. Compared with NMES, FC between ipsilesional M1 and contralesional M1/S1 were significantly strengthened during CCNMES. Additionally, significantly higher coupling strengths between ipsilesional PFC and contralesional M1/S1 were observed in the CCNMES group. Our findings suggest that CCNMES promotes the regulatory functions of ipsilesional prefrontal and motor areas as well as contralesional sensorimotor areas within the functional network in patients with stroke.

## Introduction

Stroke is a cerebrovascular disorder commonly accompanied by hemiparesis, with temporary or permanent upper extremity dysfunction reported for 50% of stroke patients (Lee et al., 2017). Loss of wrist extension is one of the most frequently persisting consequences, often limiting the ability to conduct functional activities of daily living (Zheng et al., 2019).

Neuromuscular electrical stimulation (NMES) is a conventional and effective treatment of upper extremity impairment in poststroke rehabilitation. NMES has been widely used to produce repetitive wrist extension in the paretic upper extremity in order to promote motor recovery (Wilson et al., 2016) and improve the ability of stroke patients to perform daily living tasks (Knutson et al., 2009). While NMES has been recommended as class IIa therapy for severe upper extremity hemiparesis (Winstein et al., 2016), it is essentially a passive treatment mode with limited active participation, which often evokes impatience and restlessness in patients during therapy (Shen et al., 2015).

Contralaterally controlled neuromuscular electrical stimulation (CCNMES) is an innovative NMES method originally proposed by Knutson et al. (2007). Compared with unilateral NMES, CCNMES is a bilateral and closed-loop electromyography (EMG)-controlled mode of therapy. In CCNMES, an EMG signal of voluntary wrist extension is received on the unaffected side and the corresponding electrical stimulation delivered to the hemiplegic target muscles aiming to drive symmetrical or near-simultaneous movement on the paretic side (Knutson et al., 2007, 2020). The intensity of stimulation is proportional to the degree of volitional extension of the nonparetic wrist (Shen et al., 2015). Earlier studies demonstrated that CCNMES-based neurorehabilitation exerts a superior effect to NMES in facilitating motor control in wrist extension after stroke (Shen et al., 2015; Zheng et al., 2019; Knutson et al., 2020). However, the underlying neural mechanisms remain to be resolved. In the current study, we further explored the differences in mechanisms of bilateral CCNMES and unilateral NMES with the aid of functional near-infrared spectroscopy (fNIRS).

fNIRS is a noninvasive tool which can record the relative hemodynamic response of cortical activation through the concentrations of both oxygenated hemoglobin (HbO) and deoxygenated hemoglobin (HbR). In view of its several advantages, including portability, low cost, good spatial resolution and strong anti-interference (Zhang et al., 2021), fNIRS is widely utilized in several clinical settings, especially in the field of neuroscience (Yang et al., 2019; Chen et al., 2020). fNIRS-derived functional connectivity (FC) provides an effective and powerful means to study the interactions and communications between different brain regions (Huo et al., 2019). To our knowledge, limited studies so far have focused on brain FC induced by CCNMES and NMES. Emerging evidence suggests that task-based FC provides additional insights into brain functions that cannot be elucidated under resting conditions (Vinehout et al., 2022). To address the gap in knowledge, fNIRS was effectively employed to detect hemoglobin changes in patients subjected to CCNMES or NMES therapy in the present study. To this end, 35-channel fNIRS equipment was used to measure delta HbO in the prefrontal cortex (PFC), bilateral primary motor cortex (M1), and bilateral primary somatosensory cortex (S1). Based on the collective findings, we hypothesize that bilateral CCNMES achieves increased FC across different brain areas and enhances connections between the ipsilesional and contralesional hemispheres. Data from our experiments provide a theoretical reference for understanding the mechanistic differences in cortical reorganization between CCNMES and NMES treatments.

## Methods

### Participants

Stroke patients with upper extremity hemiplegia were recruited from the Rehabilitation Medicine Center in Changzhou Dean Hospital from June 2021 to January 2022. Inclusion criteria were as follows: (1) diagnosis of hemorrhagic or ischemic stroke using computed tomography (CT) or magnetic resonance imaging (MRI), (2) 30–80 years of age, (3) subcortical and right hemisphere lesion, (4) post-stroke onset within 1 year, (5) active range of motion (AROM) of the wrist joint on the hemiplegic side less than 70°, (6) sufficient passive range of motion (PROM) of the wrist joint on the hemiplegic side, (7) intact skin and no sensory deficits on bilateral arms. Exclusion criteria were: (1) diagnosis of any clinically significant or unstable medical disorder, (2) wearing of a cardiac pacemaker, (3) skin lesions, infections, hyperalgesia, and intolerance in stimulating areas, (4) inability to follow treatment instructions due to severe cognitive and communication deficiency, and (5) lack of informed consent from patients or family members.

Experiments were conducted with the full understanding and written consent of each participant. The experimental procedure was approved by the Human Ethics Committee of Changzhou Dean Hospital (CZDALL-2021-003) and conducted in accordance with the ethical standards specified by the Helsinki Declaration of 1975 (revised in 2008). This trial was registered at ClinicalTrials.gov (ChiCTR2100048807).

### Randomization

Participants who satisfied the study criteria underwent baseline assessment by a blinded investigator prior to randomization. Enrolled patients were assigned to the NMES or CCNMES group based on a computer-generated randomization list and allocation (1:1) concealed by consecutively numbered, sealed opaque envelopes.

### Electrical stimulation system

**Figure 1:**
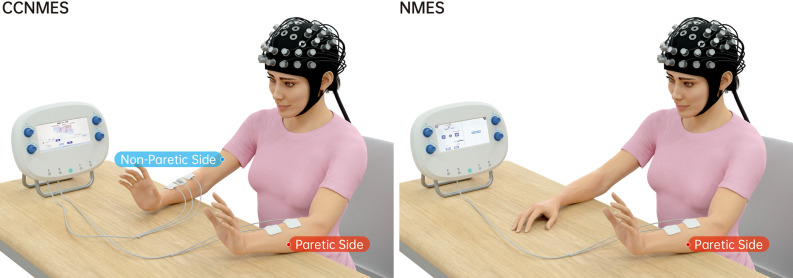
Graphical representation of contralaterally controlled neuromuscular electrical stimulation (CCNMES) and neuromuscular electrical stimulation (NMES) combined with simultaneous fNIRS monitoring.

We employed the same electrical stimulator (S4, Vishee Co., Nanjing, China) for both treatments with corresponding CCNMES and NMES treatment options for wrist extension (Figure 1).

#### CCNMES

Two 4 × 4 cm surface electrodes and one reference electrode were placed over the motor points of non-paretic forearm extensor muscles. Two 4 × 4 cm stimulatory electrodes were additionally placed over the corresponding motor points of paretic forearm extensor muscles to produce wrist extension (Gorgey et al., 2016). Prior to the experiment, subjects were asked to voluntarily extend the non-paretic wrist to 100% range of motion (ROM) and remain in this position. Maximum electromyography values were recorded. The therapist subsequently adjusted the stimulation intensity of the paretic side to evoke the same degree of wrist extension on the hemiplegia wrist without causing pain. The stimulation intensity was different among individuals, determined by the strength of contralateral forearm extensor muscles when performing voluntary wrist extension. After adjustment of the parameters, stimulation (rectangular pulse of 60 Hz, pulse width of 200 μs) was delivered with a 15 s on/10 s off cycle for 10 min.

#### NMES

Two 4 × 4 cm surface electrodes were placed over the motor points of paretic forearm extensor muscles. Stimulation intensity (pulse amplitude) was set individually to produce maximum wrist extension without inducing discomfort in the patient. After adjustment of the parameters, the stimulation was delivered with a 15 s on/10 s off cycle for 10 min. The waveform, pulse frequency and pulse width parameters were the same as those set for CCNMES.

### Functional near-infrared spectroscopy

**Figure 2:**
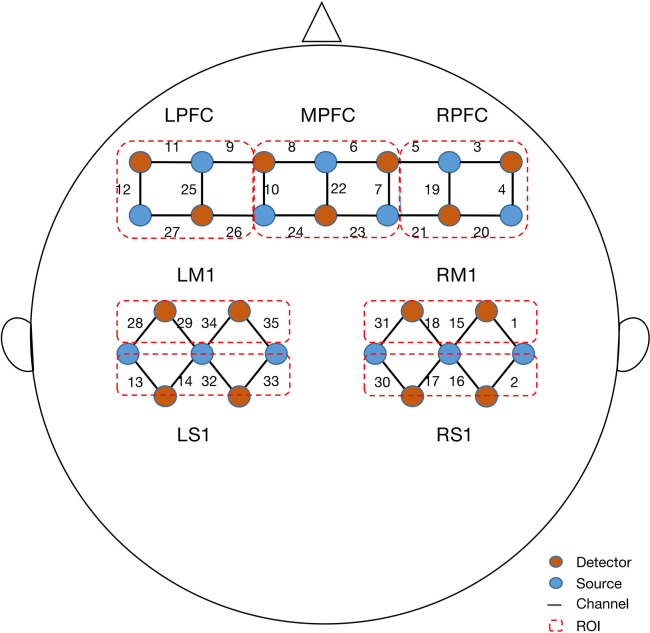
Configuration of fNIRS channels. The red dots represent detectors and the blue dots represent light sources. In total, 13 sources and 15 detectors resulted in 35 channels encompassing seven regions of interest, specifically, left prefrontal cortex (LPFC), middle prefrontal cortex (MPFC), right prefrontal cortex (RPFC), left primary motor cortex (LM1), right primary motor cortex (RM1), left primary sensory cortex (LS1), and right primary sensory cortex (RS1).

A continuous-wave fNIRS system (Nirsmart, Danyang Huichuang Medical Equipment Co, Ltd, Zhenjiang, China) at wavelengths of 730, 808, and 850 nm was utilized to measure changes in the concentrations of oxygenated hemoglobin (Δoxy-Hb) with a sampling rate of 11 Hz. A total of 35 channels set up with 16 source and 16 detector optodes were symmetrically positioned over the regions of the left prefrontal cortex (LPFC), middle prefrontal cortex (MPFC), right prefrontal cortex (RPFC), left primary motor cortex (LM1), right primary motor cortex (RM1), left primary sensory cortex (LS1), and right primary sensory cortex (RS1). The distance between the detector and source was set at 30 mm to ensure propagation to gray matter beneath the optodes. The center of the middle probe set row was placed at approximately FPz, according to the 10/20 international system. According to the standard Brodmann brain localization, all channels were divided into seven regions of interest (ROI), specifically, LPFC (9, 11, 12, 25, 26, 27), MPFC (6, 7, 8, 10, 22, 23, 24), RPFC (3, 4, 5, 19, 20, 21), LM1 (28, 29, 34, 35), RM1 (1, 15, 18, 31), LS1 (13, 14, 32, 33), and RS1 (2, 16, 17, 30; Figure 2).

### Experimental

Participants were instructed by a trained occupational therapist aiming to ensure that the experiment ran smoothly. Participants were fitted with fNIRS detection cap and seated on a chair with the stimulator placed on a desk in front. The room was lit with dim lights without noise. Each experiment involved two phases: 10-min rest and 10-min electrical stimulation task. Firstly, in the resting state, the participants were instructed to keep still with their eyes closed, relax their mind, and remain as motionless as possible (Huo et al., 2019). Then, the patients of CCNMES group were prompted by sound cues from the stimulator to repeatedly attempt to extend both wrists, hold still for 15 s when full wrist extension was achieved, and further relax for 10 s. For the patients in the NMES group, the stimulator evoked wrist extension on paretic arms with a 15 s on/10 s off cycle for 10 min.

### Data preprocessing and analysis

We adopted HbO signals as the indicator of hemodynamic response, because HbO is more sensitive to regional cerebral blood flow than HbR (Bai et al., 2020). The HomER2 toolbox (MGH-Martinos Center for Biomedical Imaging; Boston, MA, USA) in Matlab 2014a (MathWorks; Natick, MA, USA) was used for offline data preprocessing. Pre-processing procedures involved a series of steps. (1) The raw NIRS light intensity was converted to an optical density signal. (2) Motion artifact reduction algorithm (MARA) was used to detect and correct motion artifacts caused by head movements during data acquisition (parameters set as tMotion = 1 s, tMAsk = 2.0, STDEVthresh = 15.0, AMPthresh = 5.0). (3) Spline interpolation algorithm was further applied to correct motion artifacts. Following completion of artifact detection, the current detection window slid to the next detection window until the whole time series was completed. (4) Filtration: a low-pass bandpass filter between 0.01 and 0.3 Hz was applied to remove the effect of physiological noises and drifts. (5) Filtered optical density data were converted into HbO by applying the modified Beer–Lambert law (Lee et al., 2017).

### Functional connectivity

We used the 10-min resting data as our baseline data. We obtained the changes in the concentration of the oxyhemoglobin (△HbO) by taking the HbO concentration from each channel minus the baseline HbO. The 35 channels were divided into seven ROIs and the △HbO of all channels in each ROI were averaged.We then used the ROI-averaged △HbO to calculate the Pearson correlation coefficients between each ROI pairs as the functional connectivity (FC) value, which describes the linear correlation relationship of two-time domain signals using the following formula:


$$r=\frac{\sum_{i=1}^{n}\left(X_{i}-\bar{X}\right)\left(Y_{i}-\bar{Y}\right)}{\sqrt{\sum_{i=1}^{n}{\left(X_{i}-\bar{X}\right)}^{2}}\sqrt{\sum_{i=1}^{n}{\left(Y_{i}-\bar{Y}\right)}^{2}}}$$

whereby X and Y represent the time series of hemoglobin concentrations in the different ROIs, respectively, and r is the correlation coefficient, ranging from -1 to 1. Overall, 21 (7*6/2 = 21) FC values were obtained and each ROI pair (for instance, ROI1–ROI2, ROI1–ROI3) compared for both CCNMES and NMES treatment groups.

### Statistical analysis

The Shapiro-Wilk test was applied to test the variance normality and homogeneity of data at the group level. Demographics and characteristics of the stroke patients in the two groups were summarized using descriptive statistics. The categorical variables were represented by counts and percentages, and the continuous data conforming to normal distribution were represented by means with standardized deviation (SD), otherwise, represented by median and interquartile range. Two-sample *t*-tests were adopted to analyze data with normal distribution and non-parametric test (Mann–Whitney U test) applied for analysis of non-normally distributed data. For comparison of FC, we performed a two-sample *t*-test between the two groups (CCNMES vs. NMES). In order to investigate the effects of stimulation type (CCNMES/NMES) and hemispheres (left/right) on the FC, a 2 × 2 repeated-measures analysis of variance (ANOVA) was performed. A *post hoc* paired *t*-test was further applied to determine the significant differences in bilateral hemispheres between the groups.

## Results

### Clinical characteristics

Thirty-one patients were enrolled and randomly allocated to CCNMES (*n* = 16) and NMES groups (*n* = 15). The two-sample *t*-test was used to compare inter-group differences regarding age and Mann–Whitney U test applied to determine inter-group differences in terms of time post-stroke, Fugl–Meyer Assessment-upper extremity (FMA-UE) score and mini-mental state examination (MMSE). The results showed no significant differences in age, time post-stroke, FMA-UE score and MMSE between the two groups (Table 1).

**Table 1:** Demographics and characteristics of the stroke patients in the two groups.

	**CCNMES group (*n* = 16)**	**NMES group (*n* = 15)**	**t/U**	***P* value**
Age, years, mean (SD)	61.06 (11.78)	65.87 (10.32)	−1.21(t)	0.238
Gender, *n* (%)				
Male	13 (81.3%)	8 (53.3%)	-	-
Female	3 (18.8%)	7 (46.7%)	-	-
Type of stroke				
Ischemic, *n* (%)	13 (81.3%)	13 (86.7%)	-	-
Hemorrhagic, *n* (%)	3 (18.8%)	2 (13.3%)	-	-
Time post-stroke (M ± Q)	62.00 ± 106.00	40.00 ± 43.00	76.50 (U)	0.086
FMA-UE (M ± Q)	10.00 ± 31.00	4.00 ± 27.00	102.00 (U)	0.467
MMSE (M ± Q)	25.00 ± 9.00	27.00 ± 7.00	80.00 (U)	0.896

FMA-UE, Fugl–Meyer Assessment-upper extremities; MMSE, Mini-mental State Examination; U, Mann-Whitney U test; M, median; Q, Inter quartile range.

### Functional connectivity

**Figure 3:**
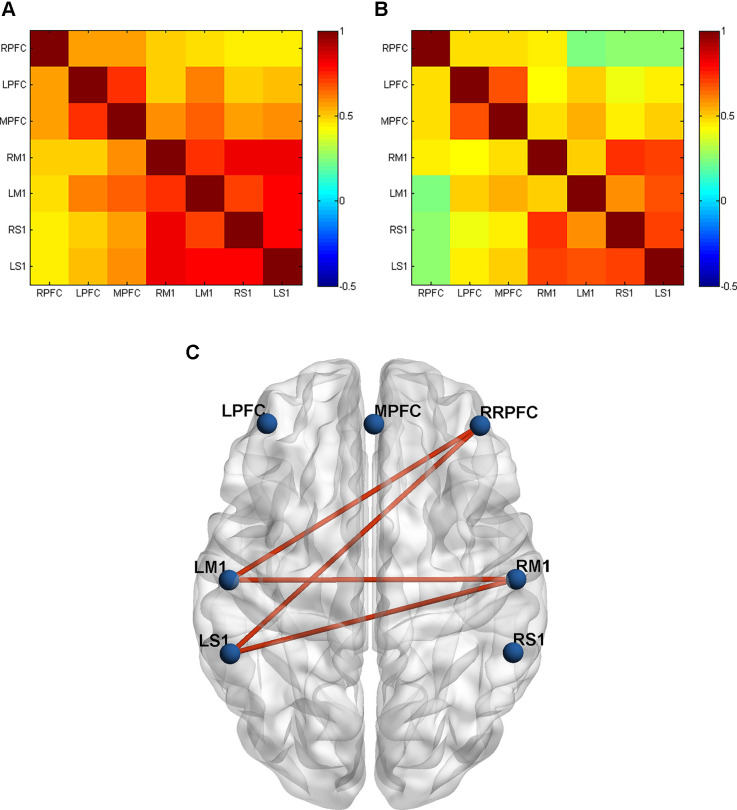
Grand-averaged correlation matrix of all ROI pairs in two groups: **(A)** CCNMES group; **(B)** NMES group. Axes represent the regions. Each channel with its correlation coefficient set at zero (the diagonal line). RPFC, right prefrontal cortex; LPFC, left prefrontal cortex; MPFC, middle prefrontal cortex; RM1, right primary motor cortex; LM1, left primary motor cortex; RS1, right primary sensory cortex; LS1, left primary sensory cortex. **(C)** The inter-group differences in actual ROIs represented by automated anatomical labelling (AAL) atlas in axial view. The blue nodes represent the seven regions of interest. The red lines represent connections with significant differences between the CCNMES group and the NMES group (all *p* < 0.05).

To explore the between-ROI connectivity characteristics, the time series of seven ROIs’ internal channels were averaged, and two-sample *t*-tests were applied to compare the differences between the CCNMES and NMES group. Compared with the NMES group, CCNMES group showed significantly increased connectivity in RM1-LM1 (*t* = -3.47, *p* = 0.002), RPFC-LM1 (*t* = -2.78, *p* = 0.009), RPFC-LS1 (*t = -2.56*, *p* = 0.016) and RM1-LS1 (*t* = -2.09, *p* = 0.046), as shown in Table 2. The grand-average correlation coefficient matrices of stroke patients in CCNMES group and NMES group were showed in Figures 3A,B, respectively, and were used to describe the between-ROIs correlation of the whole brain in each group. Figure 3C demonstrates the significant connections of ROI-pairs between the CCNMES group and the NMES group (*p* < 0.05) represented by automated anatomical labelling (AAL) atlas in axial view. Here, the right hemisphere is lesioned and left hemisphere has no lesions.

**Table 2:** The Pearson correlation coefficients between each ROI pairs in two groups.

	**NMES**	**CCNMES**	**NMES vs. CCNMES**	
	*r*	*r*	*t*	*p*
RPFC-LPFC	0.47 ± 0.21	0.57 ± 0.22	−1.31	0.2
RPFC-MPFC	0.46 ± 0.18	0.56 ± 0.24	−1.28	0.212
RPFC-RM1	0.44 ± 0.18	0.50 ± 0.22	−0.89	0.382
RPFC-LM1	0.24 ± 0.24	0.48 ± 0.21	−2.78	0.009**
RPFC-RS1	0.27 ± 0.23	0.45 ± 0.26	−1.98	0.057
RPFC-LS1	0.27 ± 0.17	0.45 ± 0.20	−2.56	0.016*
LPFC-MPFC	0.69 ± 0.13	0.72 ± 0.11	−0.57	0.576
LPFC-RM1	0.42 ± 0.22	0.49 ± 0.21	−0.93	0.36
LPFC-LM1	0.50 ± 0.25	0.62 ± 0.17	−1.44	0.161
LPFC-RS1	0.39 ± 0.16	0.49 ± 0.20	−1.49	0.146
LPFC-LS1	0.44 ± 0.21	0.51 ± 0.23	−0.87	0.391
MPFC-RM1	0.48 ± 0.16	0.59 ± 0.18	−1.69	0.102
MPFC-LM1	0.54 ± 0.18	0.65 ± 0.16	−1.7	0.101
MPFC-RS1	0.45 ± 0.22	0.56 ± 0.24	−1.27	0.214
MPFC-LS1	0.50 ± 0.19	0.60 ± 0.21	−1.26	0.216
RM1-LM1	0.50 ± 0.22	0.74 ± 0.14	−3.47	0.002**
RM1-RS1	0.72 ± 0.14	0.82 ± 0.12	−2.02	0.052
RM1-LS1	0.71 ± 0.15	0.82 ± 0.14	−2.09	0.046*
LM1-RS1	0.60 ± 0.22	0.71 ± 0.14	−1.62	0.116
LM1-LS1	0.69 ± 0.25	0.81 ± 0.14	−1.57	0.128
RS1-LS1	0.71 ± 0.17	0.79 ± 0.20	−1.16	0.256

r, Pearson correlation coefficient; *P < 0.05; **P < 0.01.

A 2 (groups) × 2 (left/right hemisphere) ANOVA of oxy-Hb signals revealed significant main effects in the lesion side (*F*_(1,29)_ = 12.579, *p* = 0.001, $\eta_{p}^{2}$ = 0.303). We observed no major interactions between the group factors (*F*_(1,29)_ = 3.10, *p* = 0.089, $\eta_{p}^{2}$ = 0.097). *Post-hoc* tests showed stronger connectivity strength of the left (contralesional) hemisphere than the right (ipsilesional) hemisphere.

## Discussion

Recovery of motor function after stroke is tightly linked to the process of reorganization of the motor system in the ipsilesional and contralesional hemisphere (Binder et al., 2021; Paul et al., 2021). To the best of our knowledge, this is the first study using the fNIRS approach to evaluate cerebral functional changes in stroke patients treated with CCNMES. Through real-time observation of the changes of FC in stroke patients under two treatment states, we found that the FC between ipsilesional M1 and contralesional M1/S1 of the stroke patients were enhanced during CCNMES treatment. Additionally, the FC between ipsilesional PFC and contralesional M1/S1 was also enhanced. Numerous studies have confirmed the positive effect of CCNMES on upper extremities of post-stroke hemiplegia (Knutson et al., 2015; Shen et al., 2015; Zheng et al., 2019), our study provide a preliminary explanation for the mechanism of its effectiveness.

In general, increased contributions from homologous regions in the contralateral hemisphere and increased connectivity between the cerebral hemispheres are considered to be one of the mechanisms of functional recovery from brain injury (Hartwigsen and Volz, 2021). Following synchronized fNIRS detection in the two states, we observed a significant higher fuctional connectivity between ipsilesional M1 and contralesional M1 under the CCNMES state. This result means that CCNMES can enhance the cortex connection between the bilateral M1 brain area than NMES. Primary motor cortex (M1) palys an important role in motor execution which is strongly lateralized to primarily distal muscles of the contralateral arm (Mohapatra et al., 2016). Upon damage of unilateral M1 and/or corresponding projections after stroke, the associated motor dysfunction may be caused (Lotze et al., 2006; Mohapatra et al., 2016). Interhemispheric M1-M1 connectivity was shown to be significantly associated with gross manual dexterity in the affected upper extremity (Peters et al., 2018). In our study, we found CCNMES can trigger more connections between ipsilesional M1 and contralesional M1 than NMES. This finding is similar to the results from Li et al.’s (2016) study in rTMS. Juan et al. (2022) also found that the 10 Hz ipsilateral rTMS group exhibited increased functional connectivity (FC) between the ipsilateral primary motor cortex (M1) and contralateral M1, which was positively correlated with motor recovery. The post-stroke motor impairment is thought to persist, in part due to an imbalance between the two hemispheres, which often explained using an “interhemispheric competition” model (Di Pino et al., 2014). Cunningham et al. (2019) using TMS to evaluate the specific impacts of CCNMES and NMES on interhemispheric inhibition (IHI). The group recorded TMS-related values before and after a 1-h session of CCNMES or NMES. They found that compared with NMES, bilateral CCNMES led to a more significant decrease in IHI between bilateral M1 areas and enhanced the output of ipsilesional motor cortices to the paretic limb. Hence, we believed that CCNMES incorporating bilateral synchronous movement can achieve decrease in intracortical inhibition within M1 (Stinear et al., 2008) and increase in interhemispheric M1-M1 connectivity (Grefkes et al., 2008), which may improve upper extremity functional recovery.

In our experiments, the fuctional connectivity between ipsilesional M1 and contralesional S1 induced by CCNMES was significantly higher than that induced by NMES. Both CCNMES and NMES essentially involve peripheral somatosensory stimulation (SS). Increased somatosensory input in the form of peripheral nerve stimulation can facilitate the recovery of motor function (Tashiro et al., 2019). fMRI-related mechanistic studies to date have demonstrated that somatosensory stimulation activates S1, secondary somatosensory cortices (S2), as well as motor-related cortices (Ibáñez et al., 1995; Backes et al., 2000; Wu et al., 2005). Neurophysiologic mapping studies additionally suggest that stimulation of cutaneous, muscle and joint afferents can drive neurons in M1 (Bolognini et al., 2016). These results provide evidence that M1 is not solely a motor structure, but rather has strong links with S1 (Bolognini et al., 2016). Knutson et al. (2012) highlighted that CCNMES produces peripheral neural activity that may be more temporally correlated with central neural activity and thus better at promoting Hebbian plasticity than NMES, consistent with our finding that association of RM1-LS1 induced by CCNMES is stronger than that by NMES. The collective results indicate that the bilateral closed-loop EMG-triggered CCNMES can effectively enhance the connection between the primary motor and sensory cortex by inducing positive intra-hemispheric coupling.

In our study, strong connections between the contralesional primary sensorimotor cortex (M1 and S1) and ipsilesional PFC were detected under CCNMES. We propose that CCNMES effectively induces cortical network connections of PFC and M1/S1, which may be attributable to CCNMES being an active mode of therapy compared to NMES, which is completely passive. NMES produces wrist movements by direct stimulation of the wrist and/or finger extensors on the paretic side, with no simultaneous effort required from the patient (Knutson et al., 2015). Conversely, CCNMES therapy creates stronger coupling of motor intention to the stimulated motor output (Knutson et al., 2009). Under CCNMES, patients are required to perform voluntary wrist extention movements to trigger electric stimulation on the paretic side (Shen et al., 2022). The intensity of electric stimulation is directly regulated by the degree of voluntary muscle contraction of the non-paretic wrist (Knutson et al., 2014). This process combines motor imagery, imitation and observation, which can mobilize motor intention to the greatest extent and promote motor relearning of participants (Shen et al., 2015). Motor relearning is accompanied by neuroplastic changes that lead to either upregulation or downregulation of regional brain activation and modulation of PFC and the motor cortex (James et al., 2013).

Significant FC were observed between bilateral hemispheres, most probably due to the fact that NMES is a unilateral while CCNMES is a bilateral mode of therapy. Numerous studies have confirmed that bilateral arm training is an advantageous approach that facilitates motor recovery in stroke patients suffering from upper extremity paresis (Waller and Whitall, 2008; Cunningham et al., 2019). In addition to benefits in the ipsilesional hemisphere, bilateral movements could engage the adaptive role of the contralesional hemisphere, especially for patients with significant corticomotor damage (Cunningham et al., 2019). In an earlier study by Whitall et al. (2011), 18 1-h sessions of bilateral therapy resulted in greater contralesional hemisphere activation, which was associated with improved arm function. Another fMRI study further validated that bilateral movements elicit unique and more significant activation of bilateral primary sensorimotor, premotor, and supplementary motor cortices relative to unilateral movements, which are amplified with therapy (Whitall et al., 2011). Notably, coupling between hemispheres was more apparent during synchronous bilateral than asynchronous movements (Cunningham et al., 2019), resulting in enhanced connectivity between bilateral hemispheres.

As sensory and motor systems within the central and peripheral nervous systems continuously interact during movements, it may be essential to combine sensory and motor training in poststroke upper limb rehabilitation (Carlsson et al., 2022). CCNMES not only involves neuromuscular electrical stimulation but also possesses various properties that promote motor function recovery, such as voluntary and bilateral symmetric movements as well as motor imaginary tasks (Knutson et al., 2009). Studies on both animals and humans indicate that active, repetitive, task-specific movement of the impaired limb is important in facilitating motor recovery after stroke (Knutson et al., 2007). The major revolutionary accomplishment of our study was measurement of brain cortex connectivity during the two different stimulation tasks. Recent research has shown that task-based functional connectivity facilitates detection of stroke-related changes not observed during the resting states (Vinehout et al., 2022), providing evidence that we detected functional connections of brain during dynamic tasks. These preliminary findings provide valuable insights into the mechanisms underlying the effects of CCNME, revealing more extensive connectivities among different brain areas with this active mode of therapy compared with NMES.

Our study has several limitations that need to be considered. First, patients were enrolled within 1 year from onset of stroke. Variations in poststroke time may lead to different reorganization patterns of the brain. Second, different sites of ischemia or hemorrhage can contribute to various brain activities. However, we constrained the lesion side of the stroke patients selected for study to the right hemisphere, which may present a considerable advantage. Finally, the brain detection tool, fNIRS, has low spatial resolution compared with fMRI and low temporal resolution compared with electroencephalography (EEG). Because we mainly aimed to detect real-time changes in cerebral blood flow under CCNMES therapy, fNIRS presented an optimal choice. Further studies involving multimodal imaging, such as fNIRS-EEG, should be conducted with strict limitation of the time poststroke as well as lesion site of the stroke to comprehensively elucidate the mechanisms underlying the observed correlations in CCNMES. At present, our results can only be interpreted within the context of the included stroke participants, i.e., those with right-sided lesions.

## Conclusion

Our study showed that CCNMES triggers sensorimotor stimulations of the affected hand sequentially involving functional reorganization of distant cortical areas after stroke. In-depth investigation of CCNMES-related changes in FC after stroke may help further our understanding of the underlying neural mechanisms. These findings provide evidence of the utility of fNIRS-derived FC in assessment of NMES and CCNMES-related changes in functional networks among the cortical areas in stroke patients.

## Data Availability Statement

The raw data supporting the conclusions of this article will be made available by the authors, without undue reservation.

## Ethics Statement

The studies involving human participants were reviewed and approved by Human Ethics Committee of Changzhou Dean Hospital. The patients/participants provided their written informed consent to participate in this study.

## Author Contributions

YShe and TW conceived and designed the study. CG, YSu, SX, RZ, JW, YZ, CK, and YShi performed the study and collected raw data. CG, MZ, YSu, and SZ analyzed the data. CG, YSu, SX, and RZ wrote the manuscript. TW and YShe helped coordinate the study and reviewed the manuscript. All authors contributed to the article and approved the submitted version.

## Funding

This study was funded by the National Key R&D Program of China (grant Nos. 2018YFC2001600 and 2018YFC2001603), the Nanjing Municipal Science and Technology Bureau (grant No. 2019060002), and the Changzhou Municipal Health Commission (grant No. QN202134).

## References

[B1] Backes W. H. Mess W. H. van Kranen-Mastenbroek V. Reulen J. P. (2000). Somatosensory cortex responses to median nerve stimulation: fMRI effects of current amplitude and selective attention. Clin. Neurophysiol. 111, 1738–1744. 10.1016/s1388-2457(00)00420-x 11018487

[B2] Bai Z. Fong K. N. K. Zhang J. Hu Z. (2020). Cortical mapping of mirror visual feedback training for unilateral upper extremity: a functional near-infrared spectroscopy study. Brain Behav. 10:e01489. 10.1002/brb3.1489 31805613 PMC6955835

[B3] Binder E. Leimbach M. Pool E. M. Volz L. J. Eickhoff S. B. Fink G. R.. (2021). Cortical reorganization after motor stroke: a pilot study on differences between the upper and lower limbs. Hum. Brain Mapp. 42, 1013–1033. 10.1002/hbm.25275 33165996 PMC7856649

[B4] Bolognini N. Russo C. Edwards D. J. (2016). The sensory side of post-stroke motor rehabilitation. Restor. Neurol. Neurosci. 34, 571–586. 10.3233/RNN-150606 27080070 PMC5605470

[B5] Carlsson H. Lindgren I. Rosen B. Bjorkman A. Pessah-Rasmussen H. Brogardh C. (2022). Experiences of SENSory relearning of the UPPer Limb (SENSUPP) after stroke and perceived effects: a qualitative study. Int. J. Environ. Res. Public Health 19:3636. 10.3390/ijerph19063636 35329318 PMC8955037

[B6] Chen W. L. Wagner J. Heugel N. Sugar J. Lee Y. W. Conant L.. (2020). Functional near-infrared spectroscopy and its clinical application in the field of neuroscience: advances and future directions. Front. Neurosci. 14:724. 10.3389/fnins.2020.00724 32742257 PMC7364176

[B7] Cunningham D. A. Knutson J. S. Sankarasubramanian V. Potter-Baker K. A. Machado A. G. Plow E. B. (2019). Bilateral contralaterally controlled functional electrical stimulation reveals new insights into the interhemispheric competition model in chronic stroke. Neurorehabil. Neural Repair 33, 707–717. 10.1177/1545968319863709 31315515 PMC6693953

[B8] Di Pino G. Pellegrino G. Assenza G. Capone F. Ferreri F. Formica D.. (2014). Modulation of brain plasticity in stroke: a novel model for neurorehabilitation. Nat. Rev. Neurol. 10, 597–608. 10.1038/nrneurol.2014.162 25201238

[B9] Gorgey A. S. Timmons M. K. Dolbow D. R. Bengel J. Fugate-Laus K. C. Michener L. A.. (2016). Electrical stimulation and blood flow restriction increase wrist extensor cross-sectional area and flow meditated dilatation following spinal cord injury. Eur. J. Appl. Physiol. 116, 1231–1244. 10.1007/s00421-016-3385-z 27155846

[B233] Grefkes C. Eickhoff S. B. Nowak D. A. Dafotakis M. Fink G. R. (2008). Dynamic intra- and interhemispheric interactions during unilateral and bilateral hand movements assessed with fMRI and DCM. Neuroimage 41, 1382–1394. 10.1016/j.neuroimage.2008.03.048 18486490

[B10] Hartwigsen G. Volz L. J. (2021). Probing rapid network reorganization of motor and language functions via neuromodulation and neuroimaging. Neuroimage 224:117449. 10.1016/j.neuroimage.2020.117449 33059054

[B11] Huo C. Xu G. Li Z. Lv Z. Liu Q. Li W.. (2019). Limb linkage rehabilitation training-related changes in cortical activation and effective connectivity after stroke: a functional near-infrared spectroscopy study. Sci. Rep. 9:6226. 10.1038/s41598-019-42674-0 30996244 PMC6470232

[B12] Ibáñez V. Deiber M. P. Sadato N. Toro C. Grissom J. Woods R. P.. (1995). Effects of stimulus rate on regional cerebral blood flow after median nerve stimulation. Brain 118, 1339–1351. 10.1093/brain/118.5.1339 7496791

[B13] James D. R. Leff D. R. Orihuela-Espina F. Kwok K. W. Mylonas G. P. Athanasiou T.. (2013). Enhanced frontoparietal network architectures following “gaze-contingent” versus “free-hand” motor learning. Neuroimage 64, 267–276. 10.1016/j.neuroimage.2012.08.056 22960153

[B14] Juan D. Yao W. Li J. Yang F. Hu J. Xu Q.. (2022). Motor network reorganization after repetitive transcranial magnetic stimulation in early stroke patients: a resting state fMRI study. Neurorehabil. Neural Repair 36, 61–68. 10.1177/15459683211054184 34711080

[B15] Knutson J. S. Fu M. J. Sheffler L. R. Chae J. (2015). Neuromuscular electrical stimulation for motor restoration in hemiplegia. Phys. Med. Rehabil. Clin. N. Am. 26, 729–745. 10.1016/j.pmr.2015.06.002 26522909 PMC4630679

[B16] Knutson J. S. Harley M. Y. Hisel T. Z. Chae J. (2007). Improving hand function in stroke survivors: a pilot study of contralaterally controlled functional electric stimulation in chronic hemiplegia. Arch. Phys. Med. Rehabil. 88, 513–520. 10.1016/j.apmr.2007.01.003 17398254 PMC3961574

[B17] Knutson J. S. Harley M. Y. Hisel T. Z. Hogan S. D. Maloney M. M. Chae J. (2012). Contralaterally controlled functional electrical stimulation for upper extremity hemiplegia: an early-phase randomized clinical trial in subacute stroke patients. Neurorehabil. Neural Repair 26, 239–246. 10.1177/1545968311419301 21875892 PMC3526819

[B18] Knutson J. S. Harley M. Y. Hisel T. Z. Makowski N. S. Chae J. (2014). Contralaterally controlled functional electrical stimulation for recovery of elbow extension and hand opening after stroke: a pilot case series study. Am. J. Phys. Med. Rehabil. 93, 528–539. 10.1097/PHM.0000000000000066 24508938 PMC4029922

[B19] Knutson J. S. Hisel T. Z. Harley M. Y. Chae J. (2009). A novel functional electrical stimulation treatment for recovery of hand function in hemiplegia: 12-week pilot study. Neurorehabil. Neural Repair 23, 17–25. 10.1177/1545968308317577 18812432 PMC3067057

[B20] Knutson J. S. Makowski N. S. Harley M. Y. Hisel T. Z. Gunzler D. D. Wilson R. D.. (2020). Adding contralaterally controlled electrical stimulation of the triceps to contralaterally controlled functional electrical stimulation of the finger extensors reduces upper limb impairment and improves reachable workspace but not dexterity: a randomized controlled trial. Am. J. Phys. Med. Rehabil. 99, 514–521. 10.1097/PHM.0000000000001363 32167957 PMC9131391

[B21] Lee M. J. Lee J. H. Koo H. M. Lee S. M. (2017). Effectiveness of bilateral arm training for improving extremity function and activities of daily living performance in hemiplegic patients. J. Stroke Cerebrovasc. Dis. 26, 1020–1025. 10.1016/j.jstrokecerebrovasdis.2016.12.008 28162905

[B22] Li J. Zhang X. W. Zuo Z. T. Lu J. Meng C. L. Fang H. Y.. (2016). Cerebral functional reorganization in ischemic stroke after repetitive transcranial magnetic stimulation: an fMRI study. CNS Neurosci. Ther. 22, 952–960. 10.1111/cns.12593 27421949 PMC6492855

[B231] Lotze M. Markert J. Sauseng P. Hoppe J. Plewnia C. Gerloff C. (2006). The role of multiple contralesional motor areas for complex hand movements after internal capsular lesion. J. Neurosci. 26, 6096–6102. 10.1523/JNEUROSCI.4564-05.2006 16738254 PMC6675223

[B230] Mohapatra S. Harrington R. Chan E. Dromerick A. W. Breceda E. Y. Harris-Love M. (2016). Role of contralesional hemisphere in paretic arm reaching in patients with severe arm paresis due to stroke: a preliminary report. Neurosci. Lett. 617, 52–58. 10.1016/j.neulet.2016.02.004 26872851

[B23] Paul T. Hensel L. Rehme A. K. Tscherpel C. Eickhoff S. B. Fink G. R.. (2021). Early motor network connectivity after stroke: an interplay of general reorganization and state-specific compensation. Hum. Brain Mapp. 42, 5230–5243. 10.1002/hbm.25612 34346531 PMC8519876

[B24] Peters D. M. Fridriksson J. Stewart J. C. Richardson J. D. Rorden C. Bonilha L.. (2018). Cortical disconnection of the ipsilesional primary motor cortex is associated with gait speed and upper extremity motor impairment in chronic left hemispheric stroke. Hum. Brain Mapp. 39, 120–132. 10.1002/hbm.23829 28980355 PMC5718970

[B25] Shen Y. Chen L. Zhang L. Hu S. Su B. Qiu H.. (2022). Effectiveness of a novel contralaterally controlled neuromuscular electrical stimulation for restoring lower limb motor performance and activities of daily living in stroke survivors: a randomized controlled trial. Neural Plast. 2022:5771634. 10.1155/2022/5771634 35069728 PMC8767388

[B26] Shen Y. Yin Z. Fan Y. Chen C. F. Dai W. Yi W.. (2015). Comparison of the effects of contralaterally controlled functional electrical stimulation and neuromuscular electrical stimulation on upper extremity functions in patients with stroke. CNS Neurol. Disord. Drug Targets 14, 1260–1266. 10.2174/1871527315666151111122457 26556084

[B232] Stinear C. M. Barber P. A. Coxon J. P. Fleming M. K. Byblow W. D. (2008). Priming the motor system enhances the effects of upper limb therapy in chronic stroke. Brain 131, 1381–1390. 10.1093/brain/awn051 18356189

[B27] Tashiro S. Mizuno K. Kawakami M. Takahashi O. Nakamura T. Suda M.. (2019). Neuromuscular electrical stimulation-enhanced rehabilitation is associated with not only motor but also somatosensory cortical plasticity in chronic stroke patients: an interventional study. Ther. Adv. Chronic Dis. 10:2040622319889259. 10.1177/2040622319889259 31798821 PMC6868577

[B28] Vinehout K. Schindler-Ivens S. Binder J. R. Schmit B. D. (2022). Task effects on functional connectivity measures after stroke. Exp. Brain Res. 240, 575–590. 10.1007/s00221-021-06261-y 34860257

[B30] Waller S. M. Whitall J. (2008). Bilateral arm training: why and who benefits. NeuroRehabilitation 23, 29–41. 10.3233/NRE-2008-23104 18356587 PMC2953420

[B29] Whitall J. Waller S. M. Sorkin J. D. Forrester L. W. Macko R. F. Hanley D. F.. (2011). Bilateral and unilateral arm training improve motor function through differing neuroplastic mechanisms: a single-blinded randomized controlled trial. Neurorehabil. Neural Repair 25, 118–129. 10.1177/1545968310380685 20930212 PMC3548606

[B31] Wilson R. D. Page S. J. Delahanty M. Knutson J. S. Gunzler D. D. Sheffler L. R.. (2016). Upper-limb recovery after stroke: a randomized controlled trial comparing EMG-triggered, cyclic and sensory electrical stimulation. Neurorehabil. Neural Repair 30, 978–987. 10.1177/1545968316650278 27225977 PMC5048487

[B32] Winstein C. J. Stein J. Arena R. Bates B. Cherney L. R. Cramer S. C.. (2016). Guidelines for adult stroke rehabilitation and recovery: a guideline for healthcare professionals from the american heart association/american stroke association. Stroke 47, e98–e169. 10.1161/STR.0000000000000098 27145936

[B33] Wu C. W. van Gelderen P. Hanakawa T. Yaseen Z. Cohen L. G. (2005). Enduring representational plasticity after somatosensory stimulation. Neuroimage 27, 872–884. 10.1016/j.neuroimage.2005.05.055 16084740

[B34] Yang M. Yang Z. Yuan T. Feng W. Wang P. (2019). A systemic review of functional near-infrared spectroscopy for stroke: current application and future directions. Front. Neurol. 10:58. 10.3389/fneur.2019.00058 30804877 PMC6371039

[B35] Zhang S. Peng C. Yang Y. Wang D. Hou X. Li D. (2021). Resting-state brain networks in neonatal hypoxic-ischemic brain damage: a functional near-infrared spectroscopy study. Neurophotonics 8:025007. 10.1117/1.NPh.8.2.025007 33997105 PMC8119736

[B36] Zheng Y. Mao M. Cao Y. Lu X. (2019). Contralaterally controlled functional electrical stimulation improves wrist dorsiflexion and upper limb function in patients with early-phase stroke: a randomized controlled trial. J. Rehabil. Med. 51, 103–108. 10.2340/16501977-2510 30671586

